# The Impact of In Vitro Digestion on the Polyphenol Content and Antioxidant Activity of Spanish Ciders

**DOI:** 10.3390/foods12091861

**Published:** 2023-04-30

**Authors:** Mari Mar Cavia, Nerea Arlanzón, Natalia Busto, Celia Carrillo, Sara R. Alonso-Torre

**Affiliations:** Área de Nutrición y Bromatología, Facultad de Ciencias, Universidad de Burgos, Plaza Misael Misael Bañuelos s/n, 09001 Burgos, Spain; nerea.arlanzon18@gmail.com (N.A.); nbusto@ubu.es (N.B.); ccarrillo@ubu.es (C.C.); salonso@ubu.es (S.R.A.-T.)

**Keywords:** apple wine, digestion protocols, antioxidants, DPPH, ABTS, FRAP, ORAC

## Abstract

Various factors can influence the polyphenol content and the antioxidant capacity of ciders, such as the apple variety, its degree of maturity, apple farming and storage conditions, and the cider-fermentation method, all of which explains why ciders of different origin present different values. In addition, digestive processes could have some effects on the properties of cider. Hence, the objective of this study is to characterize Spanish ciders in terms of their polyphenol content and antioxidant capacity and to ascertain whether those same properties differ in digested ciders. In total, 19 ciders were studied from three different zones within Spain: Asturias (A) (10), the Basque Country (BC) (6), and Castile-and-Leon (CL) (3). A range of assays was used to determine the total polyphenol content and the antioxidant capacity of the ciders. In addition, a digestive process was simulated in vitro, assessing whether the use of amylase might influence the recovery of bioactive compounds after digestion. The Basque Country ciders presented higher total polyphenol contents (830 ± 179 GAE/L) and higher antioxidant capacities (DPPH: 5.4 ± 1.6 mmol TE/L; ABTS: 6.5 ± 2.0 mmol TE/L; FRAP: 6.9 ± 1.6 mmol TE/L) than the other ciders that were studied. The in vitro digestion process, regardless of the use of amylase, implied a loss of phenolic compounds (598 ± 239 mg GAE/L undigested samples; 466 ± 146 mg GAE/L digested without amylase samples; 420 ± 115 mg GAE/L digested with amylase samples), although the variation in antioxidant activity depended on the assay chosen for its determination.

## 1. Introduction

Cider, as defined in Spanish legislation, is an alcoholic beverage resulting from the total or partial fermentation of fresh apples or their must [1]. Both cider and white wine follow very similar fermentation processes, which in the case of cider, involve alcoholic fermentation of the apples, clarification and maturation in a tank, and carbonation for sparkling ciders, before stabilization and bottling. The chemical composition of cider is mainly water and sugars (approximately 96% and 4%, respectively), small quantities of minerals and vitamins [2], and other minority substances such as phenolic compounds. These phenolic compounds are, in great measure, responsible for the antioxidant properties of cider [3], which can reduce the risk of illnesses such as hypertension, cardiovascular disease, diabetes, cancer, and inflammation [4,5].

Both the total quantity and the type of phenolic compounds found in cider depend, among other factors, on the apple variety, its degree of maturity, apple farming and storage conditions, and the cider fermentation method [3,6,7,8,9,10], for which reason ciders of different origins differ in their phenol content, and consequently, in their antioxidant properties. Accordingly, different authors have characterized the antioxidant potential of ciders of different geographic origins [9,10,11,12], although any comparison between available studies is complicated due to the variability observed in the tests employed for its determination.

It is worth highlighting that the studies available to date have been centered on assaying the antioxidant potential of fresh cider. However, any digestive effects on the functionality of this drink have not been fully investigated. Moreover, there are many studies in which changes to the content of bioactive compounds and antioxidant capacity were confirmed after the digestive process of various food matrices [13,14,15]. The digestive processes that food and drink undergo when ingested alter their pH balance, together with enzymatic action, can modify the bio-accessibility and the bio-availability of the phenolic compounds and their bioactivity [16,17,18,19,20]. The in vitro digestion models are generally the most widely employed in this sort of study. These models simulate the digestive process, including the three (oral, gastric, and intestinal digestion) stages that form part of that process, although they are usually adapted to the features of the food matrix at the center of each study. Therefore, Minekus et al. [21] observed that when simulating the digestive process in drinks without starch, it was not necessary to introduce the oral digestion phase. However, Álvarez et al. [22] observed that α-amylase, after oral and gastro-intestinal digestion of drinks, could positively influence the extraction of antioxidant compounds.

Spain is one of the main European producers of cider, total production of which in 2021 amounted to 1,046,000 hectoliters [23]. Cider production is especially centered in the Spanish autonomous regions of Asturias and the Basque Country. Various studies have been completed to date that evaluates the content and the profile of raw cider polyphenols, as well as their antioxidant capacity [3,9,12,24]. The same antioxidant assay was not, however, employed in all of those studies, nor was the in vitro digestion effect on that property. Hence, the objective of this study was to analyze the total polyphenol content and the antioxidant capacity of ciders produced in Asturias, the Basque Country, and Castile-and-Leon (neighboring the first two regions, whose production of cider is very limited), to study whether there were differences between them. In addition, it was a question of evaluating the stability of the antioxidant capacity and the total polyphenol content after the cider had undergone in vitro digestion, observing whether the oral digestion phase (effect of α-amylase) might influence those parameters in a significant manner. Finally, the assay results were studied to assess whether the effect of the different assays was a factor in the different antioxidant capacities recorded after in vitro digestion.

## 2. Materials and Methods

### 2.1. Reagents

The following reagents and enzymes were used: Folin–Ciocalteau reagent, gallic acid, ABAP (n-hexane, 2,2′-azobis-2-amidinopropane), ABTS (2,2′-Azinobisbis(3-ethylbenzothiazoline-6sulfonic acid) diammonium salt), DPPH (2,2-diphenyl-1picrylhydrazyl), TPTZ (2,4,6-Tris(2-pyridyl)-s-triazine), Trolox (6-Hydroxy-2,5,7,8-tetramethylchroman-2-carboxylic acid), iron (III) chloride-6-hydrate (FeCl_3_·6H_2_O), potassium peroxodisulphate (K_2_S_2_O_8_), and fluorescein were supplied by Sigma-Aldrich Co. α-amylase extracted from human saliva A1031, pepsin from porcine gastric mucosa P7000, pancreatin from porcine pancreas P1750, and bile salts B8756 were supplied by Sigma-Aldrich Co for the simulated digestion process. Other commonly used laboratory reagents of analytical grade were purchased from Merck KGaA (Darmstadt, Germany), Panreac (Barcelona, Spain), and Sigma-Aldrich Co. (St. Louis, MO, USA).

### 2.2. Samples

The samples used for the development of this study were 19 ciders from three different geographical areas, such as Asturias (10), the Basque Country (6), and Castile-and-Leon (3), purchased in shopping malls and craft markets. The number of samples from the region of Castile-and-Leon was very limited, as only three cider producers were found.

Once opened, the samples were separated into three fractions: one fraction was analyzed as “UnDigested” (UD) cider, another fraction underwent “in vitro Gastro-Intestinal Digestion” (GID), and the third fraction underwent “in vitro Oral and Gastro-Intestinal Digestion” (OGID).

These fractions were divided into aliquots and stored while frozen at −21 °C until their analysis. The analysis of total phenol content and antioxidant capacity was performed on each fraction and extract.

### 2.3. In Vitro Digestion

Two in vitro digestion processes were performed:

- Gastro-intestinal digestion (GID): simulating the digestive process within the stomach and in the intestinal lumen and preparing GID samples with additions of pepsin, pancreatin, and bile salts.
- Oral, Gastric, and Intestinal Digestion (OGID): simulating the oral, gastric, and intestinal digestive process and preparing the OGID samples with additions of α-amylase, pepsin, pancreatin, and bile salts. The enzymatic digestive process was prepared following the protocol of Miller et al. [25], adapted by Rufián-Henares and Delgado-Andrade [26], and modified by Pastoriza et al. [27] to include the preliminary oral digestion phase. Briefly, the physiological extraction was performed in 3 stages: oral, gastric, and intestinal. In the first place, oral digestion was simulated. Therefore, salivary α-amylase was added to 10 mL of cider, which was incubated and continuously stirred in a vertical rotary shaker (STUART ROTATOR SB3) inside an incubator (Heratherm THERMO SCIENTIFIC) for half an hour at 37 °C. Subsequently, the pH was adjusted to 2, and a pepsin solution was added in the phase simulating gastric digestion. Once again, the sample was incubated and continuously stirred for 2 h at 37 °C. The last phase simulated the digestive processes of the small intestine. The pH was adjusted to 5.3, then the pancreatin solution and bile salts were added, and the pH was once again adjusted to 7.5. The samples were incubated and stirred for 2 h at 37 °C. After completing digestion, the samples were placed in a bath at a temperature of 100 °C for 4 min and then ice-cooled. Finally, samples were centrifuged (HERAEUS Megafuge 16R) at 5500× *g* for 1 h at 4 °C. Afterward, a soluble, bio-accessible fraction was obtained that was stored at −30 °C until its analysis. No insoluble fraction remained; therefore, the subsequent fermentation phase was not carried out.

The OGID samples underwent the complete process, whereas the digestive phase with α-amylase was omitted in GID samples.

### 2.4. Determination of Total Polyphenols

Total phenolic content was measured using the Folin–Ciocalteau method [28]. An amount of 100 µL of previously diluted cider was mixed with 500 µL of Folin reagent and 400 µL of Na_2_CO_3_. Subsequently, the samples were incubated for 120 min at room temperature in the dark. Then, absorbance was read using a spectrophotometer VWR UV 6300-PC at 760 nm against a blank.

For quantification, a calibration curve was prepared using gallic acid as standard. The results were expressed in mg of equivalents of gallic acid/L cider (mg GAE/L cider).

The final content of total phenols in each type of sample (UD, GID, OGID) was corrected by its corresponding blank.

### 2.5. Chemical Methods to Determine Antioxidant Capacity

The antioxidant capacity was evaluated with different methods. In all cases, the corresponding blanks were taken into account in the calculations.

#### 2.5.1. DPPH Assay

In the DPPH assay, 2,2-diphenyl-1-picrylhydrazyl (DPPH) is used as a free radical, which produces a decrease in absorbance at 525 nm when reduced by an antioxidant [29].

The DPPH stock solution was prepared by dissolving 20 mg of DPPH in 100 mL of methanol. A working solution was obtained by diluting the stock solution until an absorbance between 0.7 and 0.8 versus methanol was obtained. Then, 20 µL of pre-diluted cider was mixed with 980 µL of the DPPH working solution and incubated for 120 min at room temperature in the dark. Absorbance was read using a spectrophotometer VWR UV 6300-PC at 525 nm to measure its radical scavenging potential.

For quantification, a calibration curve was prepared using Trolox as standard, and the results were expressed in millimole equivalents of Trolox/L cider (mmol TE/L cider).

#### 2.5.2. ABTS Assay

The ABTS assay is based on the oxidation of ABTS (2,2′azinobis-(3-ethylbenzothiazoline-6 sulfonic acid) diammonium salt by K_2_S_2_O_8_ to form the ABTS radical, which is reduced in the presence of hydrogen donor antioxidants [30].

First, a stock solution of ABTS•+ (7 mM) was prepared with K_2_S_2_O_8_ 12–16 h before use. The ABTS working solution was obtained by dilution of the stock solution up to an absorbance read of 0.75–0.80 at 734 nm against water. Then, 20 µL of pre-diluted cider was mixed with 980 µL of the ABTS working solution and incubated for 30 min at room temperature in the dark. Finally, absorbance was read using a spectrophotometer VWR UV 6300-PCat 734 nm.

For quantification, a calibration curve was prepared using Trolox as standard, and the results were expressed in millimole equivalents of Trolox/L cider (mmol TE/L cider).

#### 2.5.3. FRAP Assay

The FRAP assay (ferric reducing antioxidant power) is a measure of the capacity of an antioxidant substance to reduce Fe^3+^ to Fe^2+^, which is less antioxidant. The colorless ferric-2,4,6-tripyridyl-s-triazine complex (TPTZ) was reduced to a ferrous complex colored by an antioxidant within an acid medium [31].

The FRAP solution was prepared by mixing, at a ratio of 1:1:10 (*v*:*v*:*v*), 10 mM TPTZ solution (in 40 mM HCl), 20 mM FeCl₃ solution, and 300 mM sodium acetate buffer (pH 3.6). Then, 20 µL of cider, previously diluted, was mixed with 980 µL of the FRAP solution and incubated for 30 min at 37 °C in the dark. Finally, absorbance was read using a spectrophotometer VWR UV 6300-PC at 593 nm against a blank.

A calibration curve was prepared using Trolox as standard, and the results were expressed in millimole equivalents of Trolox/L cider (mmol TE/L cider).

#### 2.5.4. ORAC Assay

The ORAC assay (Oxygen Radical Absorbance Capacity) is a measure of the capacity of antioxidant compounds to donate a hydrogen atom that blocks free radicals. The artificial radical ABAP (2,2′-Azobis-(2-aminopropane)-dihydrochloride) oxidizes fluorescein so that it loses its fluorescence. Therefore, the antioxidant substances present in the sample reduce fluorescence decrease. Trolox was used as the standard for this determination [32].

The procedure was performed with a 96-well plate. Control samples, samples with the antioxidant (Trolox), and others with the previously diluted samples were prepared. Subsequently, fluorescein and phosphate buffer pH 7.4 were added, and emission at 511 nm (λ_exc_ = 493 nm) was read in a microplate reader (Cytation 5 Cell Imaging Multi-Mode Reader BIOTEK Instruments). Afterward, ABAP was added to a final volume of 200 µL. Then, fluorescence emission at 511 nm (λ_exc_ = 493 nm) was measured every 4 min until it reached zero, at a constant and equal temperature of 37 °C.

The protective antioxidant effect was measured by calculating the difference of areas under the fluorescence curves (AUC) over time between each sample and the control sample (without antioxidants). The AUC was calculated using the Origin program, v. 75E. The results were expressed as millimole equivalents of Trolox/L cider (mmol TE/L cider).

### 2.6. Percentage Recovery

The percentage recovery rates of the phenolic compounds and the compounds with antioxidant activity that were obtained after digestion were calculated using Equation (1):

Recovery (%) = AD/BD × 100(1)

AD is the total polyphenol content or antioxidant activity quantified in each sample after digestion.

BD is the total polyphenol content or antioxidant activity quantified in each sample before digestion.

### 2.7. Statistical Analysis

All tests and analyses were conducted in triplicate. The results were expressed as mean ± standard deviation. It was verified that the parameters followed a normal distribution. The existence of significant differences between different parameters or samples was evaluated using a *t*-test or a one-way ANOVA test (*p* < 0.05) followed by a least significant difference (LSD) post hoc test for multiple comparisons. Likewise, the Pearson correlation test was used to study whether there were relationships between the different parameters that were analyzed (*p* < 0.05). A multivariate principal component (PC) analysis was also performed.

Statgraphics Centurion 19 was used to process the statistical treatment of the data (Statgraphics Technologies, Inc., The Plains, VA, USA).

## 3. Results and Discussion

### 3.1. Polyphenol Content and Antioxidant Activity in Ciders from Different Origins

**Figure 1:**
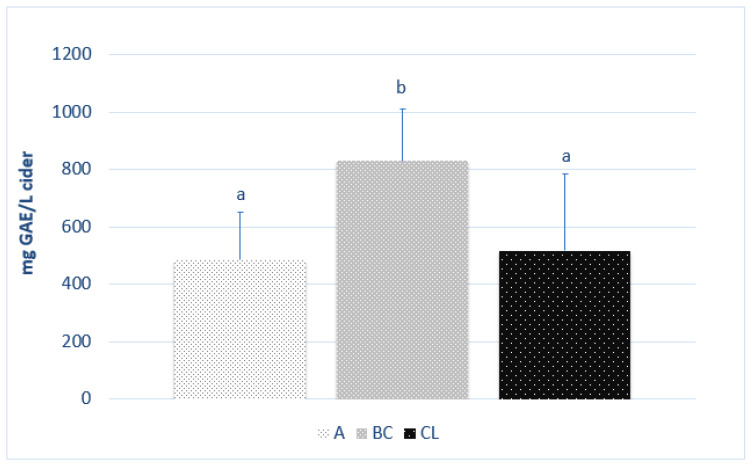
Total polyphenol content of each cider of different origin (*n* = 3). Different letters refer to significant differences (*p* < 0.05, ANOVA + LSD) between results for each origin: Asturias (A); Basque Country (BC); Castile-and-Leon (CL).

The average polyphenol content of each cider is shown in Figure 1 by its origin. The values ranged between 248 and 794 mg GAE/L for the Asturian ciders, 655 and 1153 mg GAE/L for the Basque ciders, and 338 and 826 mg GAE/L for the Castile-and-Leon ciders. The ciders produced in the regions of Asturias and Castile-and-Leon presented no statistical differences between each other, unlike the ciders prepared in the Basque Country (*p* < 0.05), which showed higher contents of polyphenols.

The values reported in other studies were somewhat higher than our own, both for Asturian ciders, with values between 447 and 1180 mg GAE/L [3], and for Basque ciders, with values between 400 and 3600 mg GAE/L [12]. No studies on ciders prepared in Castile-and-Leon were found. Among the results for ciders produced in other countries, values between 224 and 644 mg GAE/L were found for Croatian ciders [33], and an average value of 336 ± 120 mg GAE/L, representing the sum of the individual compounds obtained by HPLC in ciders from South Estonia [7].

These differences between ciders of varied geographic origins may be due to different factors, such as apple variety, degree of maturity, apple farming and storage conditions, and the cider fermentation method [9,12,22,34]. The process of preparing the Asturian ciders was very similar to the process followed in the Basque Country [24], for which reason the differences found in this study between the ciders of both origins might be due to other factors. Fermentation during the cider-making process contributes in great measure to modify the quantity and the profile of the polyphenols that each apple variety brings to the cider [34]. Rodríguez et al. [24] observed that when arable soils are fertilized at moderate or low levels with nitrogen, as is a traditional practice in apple farming in Asturias, then high levels of phloridzin accumulate within the fruit that may then be absorbed in the cider. However, the apples are exposed to the open air for two or three days during pressing in the process of producing Asturian cider, causing high levels of oxidation, which leads to the formation of large polymers, affecting polyphenol bio-accessibility. Likewise, many of the polyphenols are absorbed in the pulp of the apple, which explains the subsequent reduction in its antioxidant activity in comparison with fresh apples [35].

If we compare the above data with information found in the bibliography for other vegetable-based fermented alcoholic beverages, we find that the polyphenol content in the case of red wine varies between 1600 mg GAE/L [36] and 3500 mg GAE/L [37], values that are very much higher than those found in cider. In the case of white wine and beer, very similar values to cider have been found: 210 mg/L in white wine and 560 mg GAE/L in beer [34].

**Figure 2:**
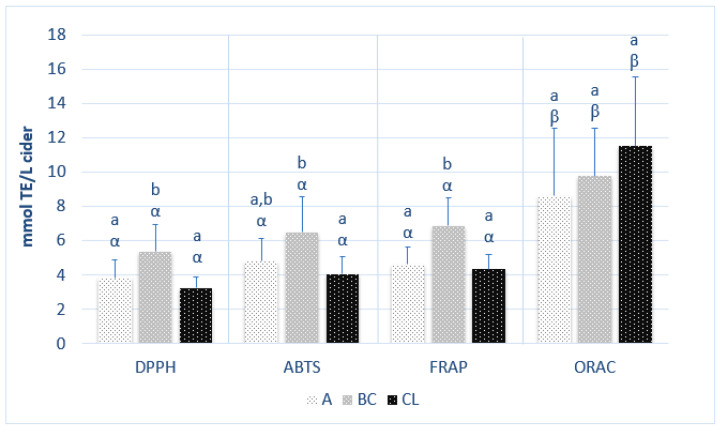
Antioxidant capacity of the ciders of different origins (*n* = 3) in each type of assay. Different Latin letters point to significant differences (*p* < 0.05, ANOVA + LSD) between results of each assay for the different origins. Different Greek letters point to significant differences between ANOVA results of each assay for the same origin (*p* < 0.05). Asturias (A); Basque Country (BC); Castile-and-Leon (CL).

In Figure 2, the average values of the antioxidant activity of the ciders of different origins are shown in relation to each of the different methods: DPPH, ABTS, FRAP, and ORAC.

According to the DPPH assay, the values for antioxidant activity were within the ranges of 2.35–5.73 mmol TE/L in Asturian ciders, 3.74–7.78 mmol TE/L for the Basque ciders, and 2.67–3.86 mmol TE/L for the Castile-and-Leon ciders. The values were somewhat higher when antioxidant activity was measured with the ABTS assay, ranging between 3.25 and 7.58 mmol TE/L in the Asturian ciders, 5.02 and 10.32 mmol TE/L in the Basque ciders, and 2.98 and 5.06 mmol TE/L in the Castile-and-Leon ciders. When using the FRAP assay, the values varied between 3.47 and 6.84 mmol TE/L in Asturian ciders, 5.35 and 9.75 mmol TE/L for the Basque ciders, and 3.30 and 4.92 mmol TE/L for the Castile-and-Leon ciders. The ORAC assay yielded the best results, varying between 4.84 and 17.08 mmol TE/L in the Asturian ciders, 6.00 and 13.13 mmol TE/L in the Basque ciders, and 7.84 and 15.87 mmol TE/L in the Castile-and-Leon ciders. It was observed that the antioxidant capacity of the ciders was slightly different according to the assay employed for its determination, coinciding with the observations of other studies on other food product matrices [14,15,38,39,40]. Although no statistically significant differences were observed between the results of the DPPH, the ABTS, and the FRAP assays, there were significant differences for ORAC.

The differences between the ciders of diverse origins were observed to vary depending on the assay that was used. The higher average values of the Basque ciders tested with the DPPH, the ABTS, and the FRAP methods were statistically different (*p* < 0.05) from the average values of the Asturian (except in the case of ABTS) and the Castile-and-Leon ciders. During the process of preparing the Basque ciders, 200 mg/L of ascorbic acid is usually added to avoid browning, and 20 mg/L of potassium metabisulfite is added as a conservative [12]; these are compounds that could, among other factors, be the cause of this greater antioxidant capacity. However, according to the ORAC assay, the Castile-and-Leon ciders presented higher average values than the ciders from the other two geographic origins, although there were no statistical differences between the three groups.

According to Álvarez et al. [22], in their study on fruit juice, the FRAP values were, in general, always higher than the ABTS values, as was also observed in this study for the Basque and the Castile-and-Leon ciders. Their results suggested that the reductive capacity of the antioxidants present in these ciders was stronger than their anti-radical activity. It should be taken into account that the different pH levels of the reactive media (strongly acidic according to the FRAP assay) might favor reductive activity. According to Floegel et al. [41], the ABTS test yielded a better estimation of the antioxidant activity of fruit and vegetables and alcoholic beverages than the DPPH (which in this study yielded the lowest results), for which reason, it was the assay that was chosen to estimate the antioxidant capacity of the ciders.

There are no studies to the best of our knowledge in which all four methods are used to assay the antioxidant capacity of ciders. For example, Picinelli et al. [3] used the DPPH and the FRAP assays for Asturian ciders, noting values of 2.9 ± 0.7 mM and 5.4 ± 1.3 mM, respectively. However, those results cannot be compared with ours as they are expressed in vitamin C equivalents of antioxidant capacity. In the case of single-variety Basque ciders that underwent the ABTS assay [12], antioxidant activity values between 6.2 ± 0.4 mmol TE/L for the Urbeti-Haundi variety and 26.0 ± 1.0 mmol TE/L for the Merabi variety were found, the latter very much higher to those observed in our study, in which the ciders were produced with mixtures of apple varieties. The same authors noted antioxidant activity values with the FRAP assay that were similar to those of this study, between 2.7 ± 0.1 mmol TE/L for the Urbeti-Haundi variety and 12.2 ± 0.5 mmol TE/L for the Merabi variety. FRAP values were lower than the ABTS values, unlike what was observed in this study. In the case of Turkish ciders, Budak et al. [11] noted higher values than in this study (an average value of 13.27 mmol TE/L) using the ABTS assay; however, the ORAC values were similar to the values of the Spanish ciders (9.84 mmol TE/L).

**Figure 3:**
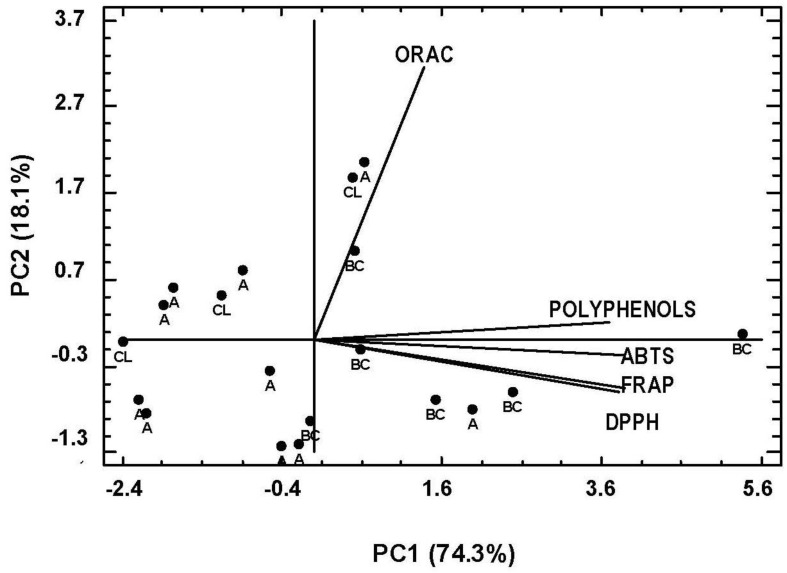
Principal component analysis (score and loading) of ciders of different origin: Asturias (A); Basque Country (BC); Castile-and-Leon (CL).

A PC analysis was conducted with the purpose of studying whether it was possible to differentiate between the ciders of the three geographic origins on the basis of the parameters under study (Figure 3). Two components were sufficient to explain 93.1% of the variance. A separation between the Asturian and the Basque ciders was observed, with most of the Asturian samples found on the left-hand side of the PC1 and the Basque samples on the right-hand side. The Castile-and-Leon samples were scattered between those two groups. The origin of the ciders can therefore have a differentiating effect on their total polyphenol content and antioxidant capacity of the ciders.

If the antioxidant activity of the ciders is compared with some other fermented drinks, the same tendency as with total polyphenols was observed. In the case of red wine, regardless of the assay that was employed, higher values were obtained than the values of the ciders that figure in this study: 12.14 mmol TE/L [36] and 18.7 mmol TE/L with the ABTS assay [37]; 25 mmol TE/L [42] with the FRAP assay; and 25.7 mmol TE/L with the ORAC assay [37]. Very scattered values were found in the case of beer, depending on the assay, which were between 0.1 mmol TE/L in the ABTS assay and 1.1 mmol TE/L in the FRAP assay [36], all very much lower values than those of the ciders.

The antioxidant activity of ciders reported in the various studies published to date [3,9,12,22,34] was measured on original samples after centrifugation and/or filtering. However, the real antioxidant activity of the drink cannot be assayed in this way because the bio-accessibility of the polyphenols and the other antioxidant compounds may be modified after the digestion process. In this study, it was decided to apply an in vitro digestion process in order to determine the extent to which the total polyphenol content and the antioxidant capacity were affected. After carrying out the simulated digestion, all that remained was a soluble fraction, in which the relevant parameters were determined.

### 3.2. Effect of In Vitro Digestion on the Phenolic Content of Ciders

**Figure 4:**
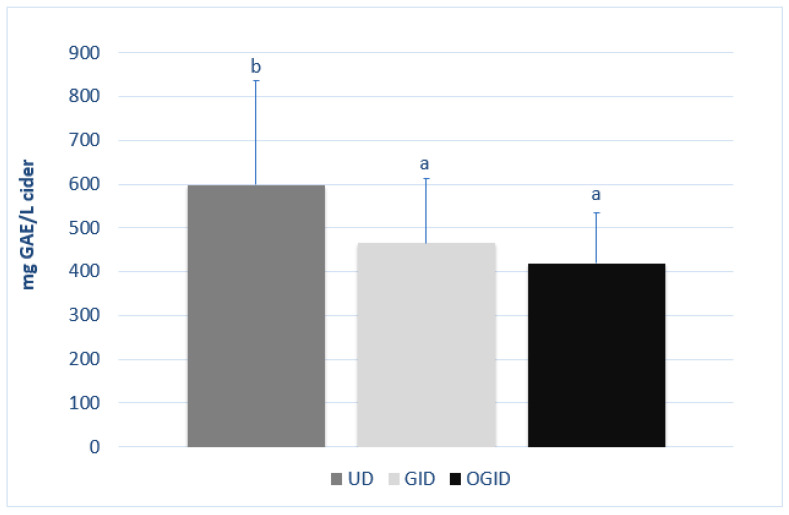
Total polyphenol content of each digested and undigested cider sample (*n* = 3). Different letters indicate significant differences (*p* < 0.05, ANOVA + LSD) between results of each sample: undigested samples (UD); gastro-intestinal digested samples (GID); oral and gastro-intestinal digested samples (OGID).

The average polyphenol contents of the digested and undigested samples are shown in Figure 4. The polyphenol content of the undigested (UD) samples ranged between 248 and 1153 mg GAE/L. In the case of the digested samples, the values ranged between 255 and 766 mg GAE/L for those that only underwent GID and between 223 and 668 mg GAE/L for the samples that underwent OGID.

The polyphenol content was significantly reduced (*p* < 0.05) after digestion. A somewhat higher liberation of polyphenols without amylase than with amylase was observed, although there were no statistical differences between both digestive processes.

If we study the ciders on an individual basis, the loss of total polyphenols after in vitro digestion may be seen to occur in most of the 19 samples under analysis (Table 1). An increase in polyphenols was only observed in four of them, both after digestion with and without amylase. All of them were Asturian ciders, some of which had the lowest polyphenol content. On the contrary, the highest losses were on the whole for the Basque ciders, which, in general, presented the highest content of polyphenols.

This reduction in the recovery of polyphenols after the in vitro digestive process has been observed by other authors in different vegetal matrices [20,43,44,45] and even in fruit wines [46]. However, an increase was observed in other food matrices [43,47,48]. It may therefore be observed that the type of sample employed, including the variety of apple, influences the liberation of polyphenols following in vitro digestion [49,50]. A variation in the polyphenol content after in vitro digestion and some examples of the post in vitro modification of the polyphenol profiles were also observed [45,51].

Most dietary polyphenols are found in nature in their glycosylate or esterified forms, and once ingested, they can then undergo modifications during the digestive process that imply the rupture of the bonds that they maintain with those compounds, provoking their solubilization and increasing their liberation. Subsequently, these free phenols can undergo certain transformations, forming complex phenolic derivatives that cannot be detected with spectrophotometric methods [20,52]. These modifications, due to both pH changes and different enzymatic actions, can affect important aspects such as bio-accessibility and bio-activity [20,43,53,54].

In general, the larger molecules are the most stable, but if they are hydrolyzed during the in vitro digestive process, they will be transformed into other smaller ones, losing stability. Gastric digestion may be said to assist the bio-accessibility of these compounds, as greater polyphenol extraction capacities are generally observed at low pH values [54]. However, it was found in other studies that flavonoids such as quercetin or anthocyanins were susceptible to degradation under alkaline conditions (pancreatic digestion) [55,56]. The low bio-accessibility of polyphenols in the duodenal digestive phase could also be caused by the formation of mixed soluble micelles in water due to the change in pH [57].

### 3.3. Effect of In Vitro Digestion on Antioxidant Activity of Ciders

**Figure 5:**
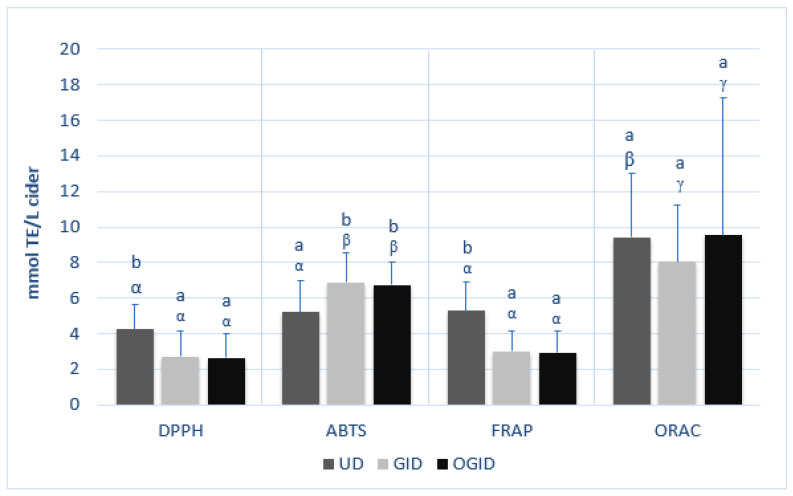
Antioxidant capacity of each digested and undigested cider sample (*n* = 3) in each type of assay. Different Latin letters point to significant differences (*p* < 0.05, ANOVA + LSD) between results of the same type of assay on the different samples. Different Greek letters point to significant differences (*p* < 0.05, ANOVA + LSD) between results of each assay on the same sample. Undigested samples (UD); gastro-intestinal digested samples (GID); oral and gastro-intestinal digested samples (OGID).

The antioxidant capacity of the ciders was assayed with different methods, and likewise, the effect of two digestions: one only gastro-intestinal and the other oral and gastro-intestinal (Figure 5).

The observable effects of the two simulated digestions of the ciders differed in accordance with the assay that was used. In the case of the DPPH test, a loss of antioxidant activity was observed after the digestions, ranging between 2.4 and 7.8 mmol TE/L in UD, 0.7 and 6.4 mmol TE/L in GID, and 0.4 and 6.4 mmol TE/L in OGID. In the case of the ABTS assay, the contrary tendency was observed as the antioxidant activity increased after digestion, yielding values between 3.0 and 10.3 mmol TE/L in UD, 4.4 and 10.7 mmol TE/L in GID, and 4.3 and 8.8 mmol TE/L in OGID. The variation in the antioxidant activity assayed with the FRAP assay followed the same trend as for DPPH, yielding values between 3.3 and 9.8 mmol TE/L in UD, 1.1 and 5.7 mmol TE/L in GID, and 0.7 and 5.3 mmol TE/L in OGID, which indicated that the samples, on average, lost reductive power following their digestion.

The in vitro digestion, therefore, provoked significative changes (*p* < 0.05) in the antioxidant capacity of the samples, at lower levels with the DPPH and FRAP assays and at higher levels with the ABTS assay; changes that were similar for both the GID and the OGID digestions.

However, according to the ORAC assay, the values of antioxidant activity ranged between 4.8 and 17.1 mmol TE/L in UD, 3.7 and 14.2 mmol TE/L in GID, and 3.8 and 34.5 mmol TE/L in OGID. There were no differences between the average corresponding values.

Although no differences between the antioxidant capacity of the UD cider samples were noted in the DPPH, the ABTS, and the FRAP assays (Figure 2), in the case of the digested samples, a different tendency was noted. In this case, the DPPH and the FRAP assays yielded similar results, both in the GID and in the OGID samples; however, significative differences existed between both those assays and the ABTS and the ORAC assays (unlike for the UD samples). These results indicated that the oral digestion step implied no modifications to the anti-radical capacity in the presence of the DPPH• radical, nor in its reductive capacity, but it made a difference to its anti-radical capacity in the presence of ABTS•+. Wootton et al. [39] indicated that the DPPH assay might underestimate antioxidant capacity after digestion due to alterations in the structure of the antioxidants that can affect their reactivity with the nitrogen radical that is formed in the DPPH assay and is biologically less relevant.

This different effect of in vitro digestion on the antioxidant activity of the ciders that were analyzed depending on the assay in use was not observed to be equal in all the ciders (Table 2). The residual antioxidant capacity after the in vitro digestion process in the DPPH assay was low, a loss that was slightly higher in the complete digestive process (OGID) but with significant differences. However, there were three (two Asturian and one Castile-and-Leon) ciders in which this capacity increased. The same was observed in a Castile-and-Leon cider assayed with the FRAP assay, where the bio-accessibility of the antioxidant compounds was improved with digestion. In the case of the ABTS assay, although the tendency was for this assay to increase the residual antioxidant activity with digestions, that tendency was reversed in the ciders with higher antioxidant values (one Asturian and two Basque ciders).

When comparing these results (Table 2) with those found in the study of polyphenols (Table 1), it was observed that the antioxidant activity determined in the DPPH and the FRAP assays better reflected the variation that the phenols underwent after the digestive process, whereas the ABTS and the ORAC assays showed variations in their antioxidant activity, unlike those found in the polyphenols. In reality, the analysis of total polyphenols refers to all the reductive compounds, for which reason, any loss of phenols logically implies a loss of reductive capacity and a lower FRAP value.

Polyphenols were among the compounds that contributed more than any others to the antioxidant capacity of the ciders, but as has been seen, the two parameters never varied in the same way after in vitro digestion. Although some antioxidants, such as polyphenols, can be destroyed during digestion, others may be formed with no variation in antioxidant activity [50,58].

Pavan et al. [58] observed increased antioxidant activity after in vitro digestion in fruit extracts, which could be related to increased polyphenol activity. This increase was also observed in other matrices, such as packaged fruit juices [59] and extracts of cloves and nutmeg [60]. Nevertheless, there are other studies where no change in the antioxidant activity of the samples was observed after in vitro digestion, such as fresh juices [59] and *Ginkgo biloba* leaves [44]. It might be due to molecular changes within some antioxidant compounds, among which the phenol groups are sensitive to pH and enzymatic attack, which can lead to variations in their chemical and functional properties [58]. The chemical structure of the food matrix in which the polyphenols are found can affect the antioxidant capacities of the vegetables that are digested, as well as possible interactions that depend on the phenolic profile [57]. Therefore, the same effect in all foods was not observed after digestion. The glycosylate and esterified phenols can hydrolyze during gastric digestion due to the low pH, thereby increasing the availability of the hydroxyl groups [61] and provoking an increase in the total polyphenol content and antioxidant activity. In contrast, the phenols that interact with the bile salts and the digestive enzymes form complex insoluble compounds and lower their original values and levels of bio-activity [62,63]. In addition, the bioactive molecules during the in vitro digestion process can form chiral enantiomers, which can lead to various reactivities in the respective fractions that are digested, modifying the antioxidant capacity, among other aspects [20]. It should be recalled when assessing antioxidant capacity, that the assays used to determine it are specific for certain structural forms [52], for which reason any interpretation of the antioxidant capacities of different tests must be treated with caution.

Another aspect that can influence the various results that were found when evaluating the effect of in vitro digestion on antioxidant capacity is the design of the digestion process. Simulated digestion with the same stages and the use of the same enzymes are not performed in all studies. For example, Álvarez et al. [22] observed that α-amylase contributed to increasing the extraction of antioxidant compounds in juices after OGID. However, no differences were found when using this enzyme in our study. Other authors found that gastric digestion increased antioxidant activity, and intestinal digestion lowered it [45]. The above results highlighted that the bioactivity of the antioxidant compounds was therefore modified by the digestion process [45,64].

**Figure 6:**
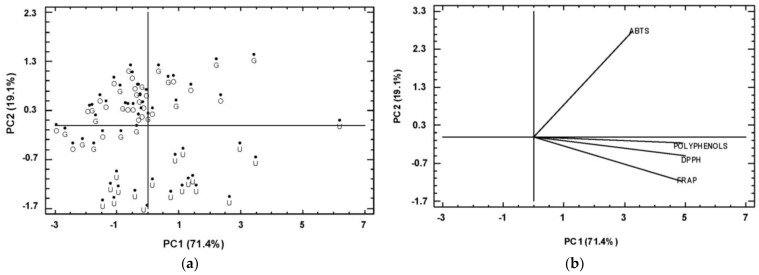
Principal component analysis, score (**a**), and loading (**b**) of ciders after the in vitro digestion processes. Undigested samples (U); gastro-intestinal digested samples (G); oral and gastro-intestinal digested samples (O).

A PCA analysis was performed to study the influence of digestive processes on the different parameters under analysis, using ABTS, FRAP, and DPPH to assay total polyphenol content and antioxidant activity in UD, GID, and OGID samples. The two initial components described a variance of 90.5% (Figure 6). Total phenols and antioxidant activity assayed with DPPH defined PC1 more than any other parameters, whereas antioxidant capacity assayed with ABTS defined PC2. The samples were separated into two clearly differentiated groups: the samples with no digestion and the samples that underwent the digestive process with no separation between the two digestions (with and without amylase). The UD samples appeared to be situated at lower values, and the digested samples (GID and OGID) at higher values than the PC2. This result is logical because the digested samples presented higher ABTS assay values, with an important weight in PC2. The majority of the digested samples appear to be grouped to the left of the PC1 in Figure 6 due to the loss of total polyphenols that was observed after the digestion process.

### 3.4. Correlation Analysis

Polyphenols are among the families of compounds present in the vegetal world that contribute most to antioxidant activity [22,65]. They have the capability to cancel the action of free radicals and to chelate the metallic ions responsible for lipid oxidation [3], although this capacity is different depending on the chemical structure of the polyphenol. A strong relationship has been found in various studies between polyphenol content and antioxidant capacity [35,41,50,66,67,68,69].

In this study, whether a correlation exists between phenolic content and antioxidant capacity observed in ciders and whether this relation may be affected by the digestive process was studied (Table 3).

A positive correlation was observed between total polyphenol content and antioxidant activity within the samples, regardless of the assay employed for their determination (DPPH, ABTS, and FRAP), with the exception of ORAC. It can be seen that the correlation coefficients were higher in the undigested samples, which indicates that the in vitro digestion process negatively affected that relation. During the process, the polyphenols are transformed by enzymatic action at the given pH, which debilitates their antioxidant capacity and, in contrast, generates other compounds, unlike polyphenols, which contribute to increasing that capacity.

Other authors have underlined the high positive correlations between polyphenol content and antioxidant capacity assayed with ABTS and DPPH [41,70,71,72] and between polyphenol contents and the FRAP values [39,70]. In the case of the Asturian ciders, Picinelli et al. [3] noted a strong positive correlation between the Folin index and the antioxidant capacity determined in both the FRAP and the DPPH assays. Both the FRAP and the ABTS tests, as well as the Folin–Ciocalteau reagent or gallic acid equivalence assay, use single electron transfer reactions that are essentially kinetic measures of the reductive capacity of an antioxidant. It is, therefore, logical that positive correlations may be observed between both tests.

The existence of these correlations depends on the food matrix, as observed by Ma and others [50]. The authors found stronger correlations in those samples with higher polyphenol contents. Those correlations highlighted that the phenols liberated during in vitro digestion played an important role in the antioxidant capacity of the samples after the digestive process [22,50].

Correlations were found between the DPPH and the ABTS assays (*p* < 0.001) (*r*^2^ = 0.8472 for UD; *r*^2^ = 0.8038 for GID; *r*^2^ = 0.7471 for OGID), ABTS and FRAP (*r*^2^ = 0.8343 for UD; *r*^2^ = 0.8235 for GID; *r*^2^ = 0.7881 for OGID), and FRAP and DPPH (*r*^2^ = 0.9608 for UD; *r*^2^ = 0.7821 for GID; *r*^2^ = 0.7575 for OGID). Transformations within the antioxidant substances were observed during the process of digestion, which weakened the degree of correlation between the different assays.

### 3.5. Limitations

The present study includes some limitations. On the one hand, only three samples were analyzed within the group of ciders from the region of Castile-and-Leon, as no other producers were found within that zone. On the other hand, total polyphenol content was determined with the Folin Ciocalteau assay so that those polyphenols that presented no reductive capacity were not determined. It is, therefore, important to study the change in the polyphenol profile with the in vitro digestion process. In addition, the assays employed to determine antioxidant activity (DPPH, ABTS, FRAP, and ORAC) were in vitro models, and not all antioxidant activities of the foods were assayed. Finally, the in vitro digestion model has only served to simulate the enzymatic actions and the pH of the digestive system, but the bioavailability of the compounds with antioxidant capacity that remained in the fractions after digestion was not evaluated.

## 4. Conclusions

In conclusion, higher polyphenol contents and antioxidant capacity were observed in the Basque ciders than in the ciders from Asturias and Castile-and-Leon.

The in vitro digestion process (regardless of the presence of the oral phase) implied a loss of bio-accessibility of phenolic compounds, although that variable in the compounds with antioxidant activity varied in accordance with the assay chosen for its determination. According to the ABTS assay, the antioxidant capacity of ciders increased after digestion. However, the DPPH and FRAP analytical results of the digested ciders showed lower antioxidant values than their raw counterparts. According to the results of the ORAC assay, in vitro digestion had no effect on antioxidant capacity.

Our results indicated that the antioxidant capacity of cider was generally underestimated with the DPPH assay, making it the least useful assay for that variable. In this investigation, the importance of using different assays and analytical methods was highlighted, especially because not one single test has yet been accepted for the measurement of total antioxidant capacity (TAC).

## Figures and Tables

**Table 1:** Recovery (%) of polyphenol content of each cider after in vitro digestion: undigested samples (UD); gastro-intestinal digested samples (GID); oral and gastro-intestinal digested samples (OGID). Asturias (A); Basque Country (BC); Castile-and-Leon (CL).

Origin	Ciders	Total Polyphenol Content UD Samples (mg GAE/L Cider)	% Recovery Polyphenols	
			GID	OGID
Asturias	A1	433 ± 38	119	122
	A2	383 ± 28	141	126
	A3	568 ± 22	65	66
	A4	585 ± 95	59	59
	A5	384 ± 17	73	68
	A6	794 ± 81	59	67
	A7	520 ± 3	75	68
	A8	248 ± 39	140	126
	A9	296 ± 48	121	136
	A10	632 ± 16	98	74
Basque Country	BC1	1153 ± 177	58	58
	BC2	655 ± 9	80	68
	BC3	861 ± 13	44	45
	BC4	802 ± 46	56	56
	BC5	679 ± 26	75	74
	BC6	830 ± 46	85	42
Castile-and-Leon	CL1	338 ± 25	76	66
	CL2	826 ± 27	93	72
	CL3	383 ± 19	92	78
Average		598 ± 239	85 ± 28 ^a^	77 ± 28 ^a^

The different superscript letters indicate significative differences (*p* < 0.05) between GID and OGID samples, according to the *t*-test results.

**Table 2:** Recovery (%) of antioxidant activity of each cider after in vitro digestion. Undigested samples (UD); gastro-intestinal digested samples (GID); oral and gastro-intestinal digested samples (OGID). Asturias (A); Basque Country (BC); Castile-and-Leon (CL).

		DPPH (mmol TE/L)	% Recovery Antioxidant Activity by DPPH		ABTS (mmol TE/L)	% Recovery Antioxidant Activity by ABTS		FRAP (mmol TE/L)	% Recovery Antioxidant Activity by FRAP		ORAC (mmol TE/L)	% Recovery Antioxidant Activity by ORAC	
Origin	Ciders	UD	GID	OGID	UD	GID	OGID	UD	GID	OGID	UD	GID	OGID
Asturias	A1	2.35 ± 0.14	126	126	3.75 ± 0.52	157	167	3.82 ± 0.30	76	72	4.84 ± 0.63	158	95
	A2	2.69 ± 0.27	153	97	4.22 ± 0.41	151	178	3.47 ± 0.48	67	62	10.36 ± 0.27	80	85
	A3	4.39 ± 0.47	33	26	4.64 ± 0.27	99	136	5.24 ± 0.08	45	41	4.96 ± 2.23	93	77
	A4	4.25 ± 0.36	34	31	5.88 ± 0.38	98	97	4.88 ± 0.09	34	32	4.89 ± 2.64	76	79
	A5	2.95 ± 0.29	24	14	3.25 ± 0.51	137	134	3.48 ± 0.09	32	21	5.53 ± 0.20	78	81
	A6	5.73 ± 0.72	37	33	7.58 ± 0.25	86	85	6.84 ± 0.08	40	39	7.87 ± 0.82	80	99
	A7	3.61 ± 0.13	58	52	3.67 ± 0.33	203	170	4.68 ± 0.42	94	47	11.85 ± 0.24	72	42
	A8	3.22 ± 0.17	124	139	3.87 ± 0.02	192	181	3.72 ± 0.34	68	80	10.08 ± 0.30	90	61
	A9	4.83 ± 0.16	40	65	4.97 ± 0.16	164	152	5.12 ± 0.43	66	70	8.66 ± 0.65	81	64
	A10	4.42 ± 0.19	93	68	6.16 ± 0.26	122	108	4.85 ± 0.10	76	66	17.08 ± 0.88	77	202
Basque Country	BC1	7.78 ± 0.05	63	50	10.32 ± 0.41	95	81	9.75 ± 0.19	51	45	13.13 ± 0.69	71	60
	BC2	4.11 ± 0.46	57	47	5.02 ± 0.31	141	146	5.63 ± 0.07	54	94	6.00 ± 2.61	99	87
	BC3	6.67 ± 0.30	20	23	7.34 ± 0.44	75	98	7.01 ± 0.08	28	35	9.06 ± 0.44	55	82
	BC4	5.46 ± 0.19	35	48	5.65 ± 0.39	123	131	7.30 ± 0.03	31	41	8.15 ± 0.51	70	124
	BC5	4.45 ± 0.29	82	87	5.37 ± 0.54	155	158	6.26 ± 0.31	61	65	9.43 ± 0.52	110	81
	BC6	3.74 ± 0.04	64	85	5.31 ± 0.09	153	128	5.35 ± 0.40	49	61	12.86 ± 0.70	111	93
Castile and Leon	CL1	2.67 ± 0.34	61	53	2.98 ± 0.02	153	143	3.30 ± 0.29	61	55	7.84 ± 0.18	132	93
	CL2	3.86 ± 0.47	166	167	5.06 ± 0.62	211	173	4.92 ± 0.20	117	104	15.87 ± 0.84	85	93
	CL3	3.27 ± 0.32	67	69	4.14 ± 0.02	140	128	4.77 ± 0.23	77	55	10.82 ± 0.64	54	224
Average		4.23 ± 1.40	70 ± 43 ^a^	67 ± 41 ^a^	5.22 ± 1.76	140 ± 38 ^a^	137 ± 31 ^a^	5.28 ± 1.61	59 ± 23 ^a^	57 ± 21 ^a^	9.44 ± 3.58	88 ± 26 ^a^	96 ± 45 ^a^

The different superscript letters indicate significative differences (*p* < 0.05) between GID and OGID samples, according to the *t*-test results.

**Table 3:** Phenolic content and antioxidant activity correlation coefficients.

Correlation Coefficient	DPPH	ABTS	FRAP	ORAC
UD	0.7627 **	0.7956 **	0.8149 **	0.3889 ^ns^
GID	0.7436 **	0.7584 **	0.6168 *	0.5995 *
OGID	0.5577 *	0.7241 **	0.6102 *	0.0701 ^ns^

Significance of correlation coefficient (Pearson): *p* < 0.001 (**); *p* < 0.05 (*); *p* > 0.05 (no significance, ns). Undigested samples (UD); gastro-intestinal digested samples (GID); oral and gastro-intestinal digested samples (OGID).

## Data Availability

The data presented in this study are available upon request from the corresponding author.

## References

[1] (2017). de 10 de Febrero, por el que se Aprueba la Norma de Calidad de las Diferentes Categorías de la Sidra Natural y de la Sidra.

[2] Moreiras O., Carbajal A., Cabrera L., Cuadrado C. (2016). Tablas de Composición de Alimentos.

[3] Picinelli A., Diñero Y., Mangas J., Rodríguez R., Suárez B. (2009). Phenolic and antioxidant composition of cider. J. Food Compos. Anal..

[4] Kumar S., Krishna Chaitanya R., Preedy V.R., Preedy V.R., Watson R.R. (2018). Assessment of antioxidant potential of dietary components. HIV/AIDS: Oxidative Stress and Dietary Antioxidants.

[5] Shahidi F., Zhong Y., Chandrasekara A., Yu L.L., Tsao R., Shahidi F. (2012). Antioxidants and human health. Cereals and Pulses: Nutraceutical Properties and Health Benefits.

[6] Lea A.G.H., Beech F. (1978). The phenolics of ciders: Effect of cultural conditions. J. Sci. Food Agric..

[7] Laaksonen O., Kuldjärv R., Paalme T., Virkki M., Yang B. (2017). Impact of apple cultivar, ripening stage, fermentation type and yeast strain on phenolic composition of apple ciders. Food Chem..

[8] Marks S.C., Mullen W., Crozier A. (2007). Flavonoid and Hydroxycinnamate Profiles of English Apple Ciders. J. Agric. Food Chem..

[9] Alonso-Salces R.M., Guyot S., Herrero C., Berrueta L.A., Drilleau J.F., Gallo B., Vicente F. (2004). Chemometric characterisation of Basque and French ciders according to their polyphenolic profiles. Anal. Bioanal. Chem..

[10] Sanoner P., Guyot S., Marnet N., Molle D., Drilleau J.P. (1999). Polyphenol profiles of French cider apple varieties (*Malus domestica* sp.). J. Agric. Food Chem..

[11] Budak H.N., Ozçelik F., Güzel-Seydim Z.B. (2015). Antioxidant activity and phenolic content of apple cider. Turk. J. Agric.-Food Sci. Technol..

[12] Zuriarrain-Ocio A., Zuriarrain J., Vidal M., Dueñas M.T., Berregi I. (2021). Antioxidant activity and phenolic profiles of ciders from the Basque Country. Food Biosci..

[13] Carrillo C., Barrio A., Cavia M.M., Alonso-Torre S.R. (2017). Global antioxidant response of meat. J. Sci. Food Agric..

[14] Carrillo C., Rey R., Hendrickx M., Cavia M.M., Alonso-Torre S.R. (2017). Antioxidant capacity of beetroot: Traditional *vs* novel approaches. Plant Foods Hum. Nutr..

[15] Zoubiri L., Bakir S., Barkat M., Carrillo C., Capanoglu E. (2019). Changes in the phenolic profile, antioxidant capacity and in vitro bioaccsessibility of two Algerian grape varieties, Cardinal and Dabouki (Sabel), during the production of traditional sun-dried raisins and homemade jam. J. Berry Res..

[16] Guo R., Chang X., Guo X., Brennan C.B., Li T., Fu X., Liu R.H. (2017). Phenolic compounds, antioxidant activity, antiproliferative activity and bioaccessibility of Sea buckthorn (*Hippophaë rhamnoides* L.) berries as affected by in vitro digestion. Food Funct..

[17] Jara-Palacios M.J., Gonçalves S., Hernanz D., Heredia F.J., Romano A. (2018). Effects of in vitro gastrointestinal digestion on phenolic compounds and antioxidant activity of different white winemaking by products extracts. Food Res. Int..

[18] Barak T.H., Celep E., Inan Y., Yesilada E. (2019). Influence of in vitro human digestion on the bioavailability of phenolic content and antioxidant activity of *Viburnum opulus* L. (*European cranberry*) fruit extracts. Ind. Crops Prod..

[19] Czubinski J., Wroblewska K., Czyzniejewski M., Górnaś P., Kachlicki P., Sigera A. (2019). Bioaccessibility of defatted lupin seed phenolic compounds in a standardized static in vitro digestion system. Food Res. Int..

[20] Qin W., Ketnawa S., Ogawa Y. (2022). Effect of digestive enzymes and pH on variation of bioavailability of green tea during simulated in vitro gastrointestinal digestion. Food Sci. Hum. Wellness.

[21] Minekus M., Alminger M., Alvito P., Balance S., Bohn T., Bourlieu C., Carrière F., Boutrou R., Corredig M., Dupont D. (2014). A standardised static in vitro digestion method suitable for food—an international consensus. Food Funct..

[22] Álvarez J., Pastoriza S., Alonso-Olalla R., Delgado-Andrade C., Rufián-Henares L.A. (2014). Nutritional and physicochemical characteristic of commercial Spanish citrus juices. Food Chem..

[23] European Cider and Fruit Wine Association. https://aicv.org/en/publications.

[24] Rodríguez R., Pinicelli A., Suárez B. (2006). Phenolic profile of asturian (Spain) natural cider. J. Agric. Food Chem..

[25] Miller D.D., Schricker B.R., Rasmussen R.R., Van Campen D. (1981). An in vitro method for estimation of iron availability from meals. Am. J. Clin. Nutr..

[26] Rufián-Henares J.A., Delgado-Andrade C. (2009). Effect of digestive process on Maillard reaction indexes and antioxidant properties of breakfast cereals. Food Res. Int..

[27] Pastoriza S., Delgado-Andrade C., Haro A., Rufián-Henares J. (2011). A physiologic approach to test the global antioxidant response of foods. The GAR method. Food Chem..

[28] Singleton V.L., Orthofer R., Lamuela-Raventos R.M. (1999). Analysis of total phenols and other oxidation substrates and antioxidants by means of Folin-Ciocalteau reagent. Meth. Enzymol..

[29] Brand-Williams W., Cuvelier M.E., Berset C. (1995). Use of a free radical method to evaluate antioxidant activity. LWT-Food Sci. Technol..

[30] Miller N.J., Diplock A.T., Rice-Evans C., Davies M.J., Gopinathan V., Milner A. (1993). A novel method for measuring antioxidant capacity and its application to monitoring the antioxidant status in premature neonates. Clin. Sci..

[31] Benzie I.F., Strain J.J. (1996). Ferric reducing ability of plasma (FRAP) as a measure of antioxidant power: The FRAP assay. Anal. Biochem..

[32] Cao G., Alessio H.M., Cutler R.G. (1993). Oxygen-Radical absorbance Capacity assay for antioxidants. Free Radic. Biol. Med..

[33] Ljevar A., Ćurko N., Tomašević M., Radošević K., Srček V.G., Ganić K.K. (2016). Phenolic composition, antioxidant capacity and in vitro cytotoxicity assessment of fruit wines. Food Technol. Biotechnol..

[34] Ye M., Yue T., Yuan Y. (2014). Evolution if polyphenols and organic acids during the fermentation of apple cider. J. Sci. Food Agric..

[35] Van der Sluis A.A., Dekker M., Skrede G., Jongen W.M.F. (2002). Activity and concentration of polyphenolic antioxidants in apple juice. 1. Effect of existing methods. J. Agric. Food Chem..

[36] Saura-Calixto F., Goñi I. (2006). Antioxidant capacity of the Spanish Mediterranean diet. Food Chem..

[37] Seeram N.P., Aviram M., Yanjun Z., Henning S.M., Feng L., Dreher M., Hber D. (2008). Comparison of antioxidant potency of commonly consumes polyphenol-rich beverages in the United States. J. Agric. Food Chem..

[38] Ou B., Huang D., Hampsch-Woodill M., Flanagan J.A.J., Deemer E.K. (2002). Analysis of antioxidant activities of common vegetables employing oxygen radical absorbance capacity (ORAC) and ferric reducing antioxidant power (FRAP) assays: A comparative study. J. Agric. Food Chem..

[39] Wootton-Beard P.C., Moran A., Ryan L. (2011). Stability of the total antioxidant capacity and total polyphenol content of 23 commercially available vegetable juices before and after in vitro digestion measured by FRAP, DPPH, ABTS and Folin–Ciocalteu methods. Food Res. Int..

[40] Wu X., Beecher G.R., Holden J.M., Haytowitz D.B., Gebhardt S.E., Prior R.L. (2004). Lipophilic and hydrophilic antioxidant capacities of common foods in the United States. J. Agric. Food Chem..

[41] Floegel A., Kim D.O., Chung S.J., Koo S.I., Chun O.K. (2011). Comparison of ABTS/DPPH assays to measure antioxidant capacity in popular antioxidant-rich US foods. J. Food Compos. Anal..

[42] Carlsen M.H., Halvorsen B.L., Holte K., Bøhn S.K., Dragland S., Sampson L., Willey C., Senoo H., Umezono Y., Sanada C. (2010). The total antioxidant content of more than 3100 foods, beverages, spices, herbs and supplements used worldwide. Nutr. J..

[43] Friedman M., Jürgens H.S. (2000). Effect of pH on the stability of plant phenolic compounds. J. Agric. Food Chem..

[44] Oliveira D., Laitmer C., Parpot P., Gill C.I., Oliveira R. (2020). Antioxidant and antigenotoxic activities of *Ginkgo biloba* L. leaf extract are retained after in vitro gastrointestinal digestive conditions. Eur. J. Nutr..

[45] Liu X., Shi J., Yi J., Zhang X., Ma Q., Cai S. (2021). The effect of in vitro simulated gastrointestinal digestion on phenolic bioaccessibility and bioactivities of *Prinsepia utilis* Royle fruits. LWT-Food Sci. Technol..

[46] Celep E., Charehsaz M., Akyüz S., Acar E.T., Yeselida E. (2015). Effect of in vitro gastrointestinal digestion on the bioavailability of phenolic components and the antioxidant potentials of some Turkish fruit wines. Food Res. Int..

[47] Wootton-Beard P.C., Ryan L. (2011). A beetroot juice shot is a significant and convenient source of bioaccessible antioxidants. J. Funct. Foods.

[48] Bhatt A., Patel V. (2015). Evaluation of Actual Antioxidant Capacity of Papaya (*Carica papaya*) Using an In Vitro Gastrointestinal Model. Int. J. Fruit Sci..

[49] He M., Zeng J., Zhai L., Liu Y., Wu H., Zhang R., Li Z., Xia E. (2017). Effect of in vitro simulated gastrointestinal digestion on polyphenol and polysaccharide content and their biological activities among 22 fruit juices. Food Res. Int..

[50] Ma Y., Gao J., Wei Z., Shahidi F. (2021). Effect of in vitro digestion on phenolics and antioxidant activity of red and yellow colored pea hulls. Food Chem..

[51] Sun D., Huang S.Q., Cai S.B., Cao J.X., Han P. (2015). Digestion property and synergistic effect on biological activity of purple rice (*Oryza sativa* L.) anthocyanins subjected to a simulated gastrointestinal digestion in vitro. Food Res. Int..

[52] Bermúdez-Soto M.J., Tomás-Barberán F.A., García-Conesa M.T. (2007). Stability of polyphenols in chokeberry (*Aronia melanocarpa*) subjected to in vitro gastric and pancreatic digestion. Food Chem..

[53] Karaś M., Jakubczyk A., Szymanowska U., Złotek U., Zielińska E. (2017). Digestion and bioavailability of bioactive phytochemicals. Int. J. Food Sci. Technol..

[54] Moyo S.M., Serem J.C., Bester M.J., Mavumengwana V., Kayitesi E. (2020). The impact of boling and in vitro human digestion of *Solanum nigrum* complex (*Black nightshade*) on phenolic compounds bioactivity and bioaccessibility. Food Res. Int..

[55] Wang J., Zhao X.H. (2016). Degradation kinetics of fisetin and quercetin in solutions affected by medium pH, temperature and co-existing proteins. J. Serb. Chem. Soc..

[56] Carrillo C., Kamiloglu S., Grootaert C., Van Camp J., Hendrickx M. (2020). Co-ingestion of black carrot and strawberry. Effects on anthocyanin stability, bioaccessibility and uptake. Foods..

[57] Caicedo-Lopez L.H., Luzardo-Ocampo I., Cuellar-Nuñez M.L., Campos-Vega R., Mendoza S., Loarca-Piña G. (2019). Effect of the in vitro gastrointestinal digestion on free-phenolic compounds and mono/oligosaccharides from *Moringa oleifera* leaves: Bioaccessibility, intestinal permeability and antioxidant capacity. Food Res. Int..

[58] Pavan V., Soriano Sancho R.A., Pastore G.M. (2014). The effect of in vitro digestion on the antioxidant activity of fruit extracts (*Carica papaya*, *Artocarpus heterophillus* and *Annona marcgravii*). LWT—Food Sci. Technol..

[59] Ryan L., Prescott S.L. (2010). Stability of the antioxidant capacity of twenty-five commercially available fruit juices subjected to an in vitro digestion. Int. J. Food Sci. Technol..

[60] Baker I., Chohan M., Opara E.I. (2013). Impact of Cooking and Digestion, In Vitro, on the Antioxidant Capacity and Anti-Inflammatory Activity of Cinnamon, Clove and Nutmeg. Plant Foods Hum. Nutr..

[61] Gonçalves S., Moreira E., Andrade P.B., Valentao P., Romano A. (2019). Effect of in vitro gastrointestinal digestion on the total phenolic contents and antioxidant activity of wild Mediterranean edible plant extracts. Eur. Food Res. Technol..

[62] Spínola V., Llorent-Martínez E.J., Castilho P.C. (2018). Antioxidant polyphenols of Madeira sorrel (*Rumex maderensis*): How do they survive to in vitro simulated gastrointestinal digestion?. Food Chem..

[63] Bayliak M.M., Burdyliuk N.I., Lushchak V.I. (2016). Effects of pH on antioxidant and prooxidant properties of common medicinal herbs. Open Life Sci..

[64] Serrano J., Goñi I., Saura-Calixto F. (2007). Food antioxidant capacity determined by chemical methods may underestimate the physiological antioxidant capacity. Food Res. Int..

[65] Albishi T., John J.A., Al-Khalifa A.S., Shahidi F. (2013). Phenolic content and antioxidant activities of selected potato varieties and their processing by-products. J. Funct. Foods..

[66] Tsao R., Yang R., Xie S., Sockovie E., Khahnizadeh S. (2005). Which polyphenolic compounds contribute to the total antioxidant activities of apple?. J. Agric. Food Chem..

[67] Vanzani P., Rossetto M., Rigo A., Vrhovsek U., Mattivi F., D’ Amato E., Scarpa M. (2005). Major phytochemicals in apple cultivars: Contribution to peroxyl radical trapping efficiency. J. Agric. Food Chem..

[68] Gullon B., Pintado M.E., Fernández-López J., Pérez-Álvarez J.A., Viuda-Martos M. (2015). In vitro gastrointestinal digestion of pomegranate peel (*Punica granatum*) flour obtained from co-products: Changes in the antioxidant potential and bioactive compounds stability. J. Funct. Foods.

[69] Lucas-Gonzalez R., Navarro-Coves S., Pérez-Álvarez J.A., Fernández-López J., Muñoz L.A., Viuda-Martos M. (2016). Assessment of polyphenolic profile stability and changes in the antioxidant potential of maqui berry (*Aristotelia chilensis* (Molina) Stuntz) during in vitro gastrointestinal digestion. Ind. Crops Prod..

[70] Gorinstein S., Haruenkit R., Poovarodom S., Vearasilp S., Ruamsuke P., Namiesnik J., Leontowicz M., Leontowicz H., Suhaj M., Sheng G.P. (2010). Some analytical assays for the determination of bioactivity of exotic fruits. Phytochem. Anal..

[71] Chun O.K., Kim D.O., Moon H.Y., Kang H.G., Lee C.Y. (2003). Contribution of individual polyphenolics to total antioxidant capacity of plums. J. Agric. Food Chem..

[72] Kim D.O., Chun O.K., Kim Y.J., Moon H.Y., Lee C.Y. (2003). Quantification of polyphenolics and their antioxidant capacity in fresh plums. J. Agric. Food Chem..

